# PARP4 deficiency enhances sensitivity to ATM inhibitor by impairing DNA damage repair in melanoma

**DOI:** 10.1038/s41420-025-02296-0

**Published:** 2025-01-30

**Authors:** Yuehua Li, Yu Liu, Jingjing Ma, Yuqi Yang, Qiao Yue, Guannan Zhu, Weinan Guo, Tianwen Gao, Qiong Shi, Chunying Li

**Affiliations:** Department of Dermatology, Xijing Hospital, Fourth Military Medical University, Xi’an, 710032 China

**Keywords:** Cancer therapy, Cell death, Prognostic markers

## Abstract

Besides the important pathogenic mechanisms of melanoma, including BRAF-driven and immunosuppressive microenvironment, genomic instability and abnormal DNA double-strand breaks (DSB) repair are significant driving forces for its occurrence and development. This suggests investigating novel therapeutic strategies from the synthetic lethality perspective. Poly (ADP-ribose) polymerase 4 (PARP4) is known to be a member of the PARP protein family. The low expression of PARP4 is significantly associated with defective DSB repair markers and poor prognosis in melanoma. Further research revealed that PARP4 plays a role in DSB repair by regulating the non-homologous end joining (NHEJ) pathway through its involvement in Ku80 mono-ADP-ribosylation. Moreover, from a synthetic lethality perspective, PARP4 expression is associated with ATM inhibitor sensitivity. Overall, our study provides new and valuable insights into the function of PARP4 and melanoma pathogenesis and suggests that ATM inhibitor may be a promising therapeutic approach for treating melanoma with low PARP4 expression.

## Introduction

Melanoma originates from the malignant transformation of melanocytes and is the most lethal skin cancer form. Advances in targeted therapy and immunotherapy have significantly improved patient outcomes [1]. However, low BRAF mutation rates in Asian populations, along with primary resistance to therapy, continue to limit therapeutic efficacy [2, 3]. Therefore, there is an urgent need to better comprehend melanoma’s pathogenesis and develop novel therapeutic strategies.

Genomic instability and abnormal DNA double-strand breaks (DSB) repair within the melanoma genome could be significant in its occurrence and progression. DSB are repaired by two principal mechanisms: homologous recombination (HR) and non-homologous end joining (NHEJ) [4]. Our previous study and other genetic-landscape studies revealed that Catalogue of Somatic Mutations in Cancer signature 3, which is associated with homologous recombination deficiency (HRD) of DSB, has been curated within acral and mucosal melanoma [5, 6]. Moreover, 33.5% of 1986 melanoma cases were identified with gene mutations from the HR pathway based on the Foundation Medicine cohort. Similarly, 41% of 1088 melanoma patients in the cBioPortal cohort exhibited mutations in HR pathway genes [7]. Additionally, indel signature 8, indicative of NHEJ-associated DSB repair defects, has been identified in most melanomas [8]. The above evidence indicates that abnormal DSB repair could potentially lead to melanoma pathogenesis. Therefore, synthetic lethal strategies could be promising therapeutic approaches.

Poly (ADP-ribose) polymerase 4 (PARP4), also called vPARP (vault PARP), is a PARP family member comprising 17 members (PARP1-16). These enzymes catalyze ADP-ribose modifications to target proteins using NAD+ as a substrate. PARP4 was identified as an interacting partner of the major vault protein (MVP), which is involved in the composition of vault complexes and plays a role in intracellular transport [9]. Our study depicted that low PARP4 expression was associated with genomic instability and defective DSB repair. Further studies have shown that PARP4 could be a prognostic biomarker in melanoma patients and participate in DSB repair through the NHEJ pathway. Moreover, inhibiting PARP4 could improve melanoma cell sensitivity to Ataxia telangiectasia mutated (ATM) inhibitors in vitro and in vivo.

## Results

### PARP4 is related to genome stability and serves as a prognostic biomarker in melanoma patients

**Fig. 1:**
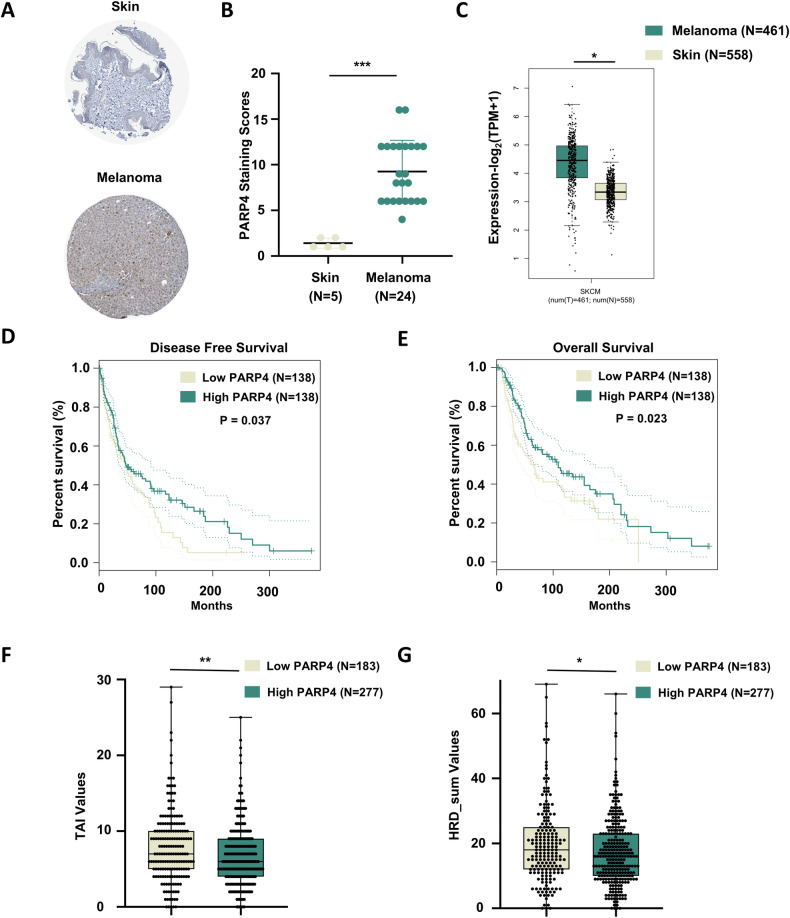
PARP4 expression of melanoma and normal skin and its correlation with genome stability and prognosis. **A** Immunohistochemical staining of the PARP4 expression level of melanoma was compared with normal skin tissue from the HPA database. **B** Quantitative analysis of PARP4 staining scores of skin and melanoma depending on the HPA database. ***P < 0.001. **C** PARP4 mRNA levels in melanoma were compared using normal tissue from the GEPIA database. *P < 0.05. **D**, **E** The association between PARP4 expression level (using 30% as a cutoff) and disease-free survival along with overall survival of patients based on the GEPIA database. The *P-*value was determined using the Log-rank test. **F**, **G** TAI and HRD_sum values of melanoma samples, divided into low PARP4 and high PARP4 expression using 40% as a cutoff, were analyzed from the TCGA database. *P < 0.05, **P < 0.01.

Firstly, we investigated PARP4 expression within melanoma tissues. According to the Human Protein Atlas (HPA) database, immunohistochemical analysis revealed significantly higher levels of PARP4 protein in melanoma tissues (*N* = 24) compared to normal skin tissues (*N* = 5) *(P* < 0.001) (Fig. 1A and B). Further analysis using the Cancer Genome Atlas (TCGA) database confirmed that PARP4 expression was notably higher in melanoma samples (*N* = 461) compared to normal skin samples (*N* = 558) (Fig. 1C). Moreover, the data of cutaneous melanoma from TCGA database indicated that low PARP4 expression was significantly related to shorter disease-free survival (DFS) (*P* = 0.037) and overall survival (OS) (*P* = 0.023) (Fig. 1D and E), indicating its significant role in the development and prognosis of melanoma. Next, we found that low PARP4 expression was significantly correlated with high telomeric allelic imbalances (TAI) values and HRD_sum values (Fig. 1F and G) in cutaneous melanoma according to the TCGA database. Both TAI and HRD scores are indicators of defective DSB repair and genome instability [10, 11]. These findings suggest that PARP4 may play a crucial role in DSB repair and contribute to melanoma pathogenesis.

### PARP4 translocates into the nucleus to participate in DSB repair when DSB occurs

To further explore the role of PARP4 in melanoma, we examined the expression status of PARP4 in melanoma cell lines. The PARP4 expression was relatively low in A2058 cells but high in FLFMM34 cells (Supplementary Fig. 1). Therefore, to explore the role of PARP4 in melanoma pathogenesis, we established siRNA and overexpression plasmid against PARP4 to secure the knockdown and overexpression of PARP4 in FLFMM34 and A2058 melanoma cells, respectively (Supplementary Fig. 2). Immunofluorescence staining revealed that PARP4 was primarily localized in the cytoplasm (Supplementary Fig. 3), which was consistent with the immunohistochemical result in melanoma tissues based on the HPA database (Fig. 1A).

**Fig. 2:**
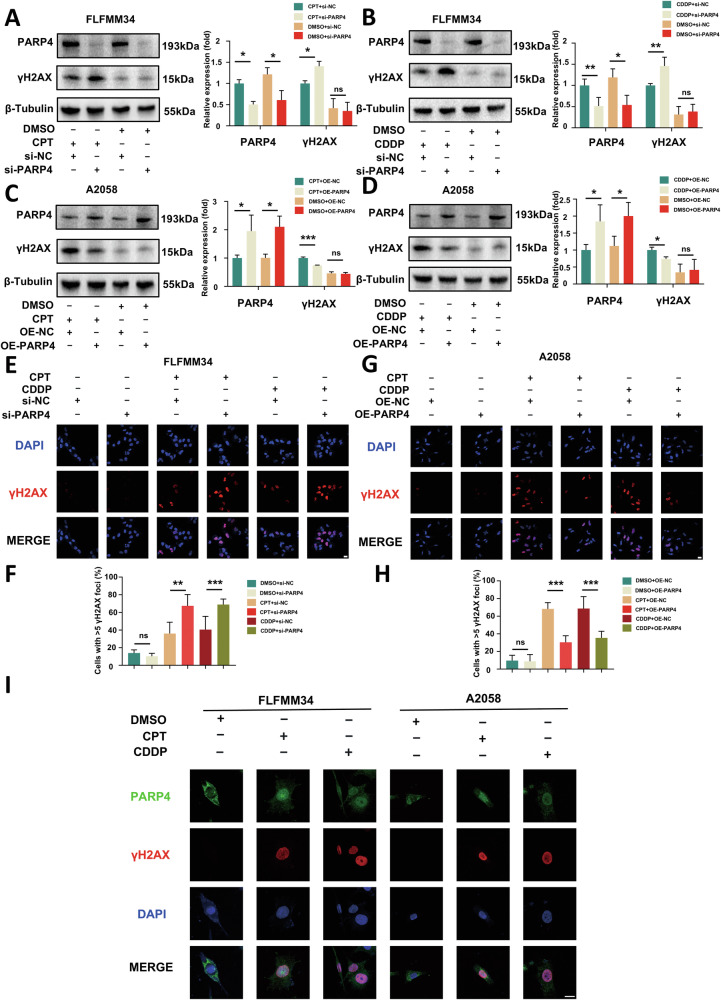
PARP4 translocates into the nucleus upon DSB to participate in DNA damage repair. **A**–**D** Western blotting and quantitative analysis of expression levels of PARP4 and γH2AX in A2058 and FLFMM34 cells when PARP4 knockdown and overexpression after treatment with 1 μM CPT, 20 μM CDDP, or DMSO for 12 h. *P < 0.05, **P < 0.01 and ***P < 0.001. ns, nonsignificant. **E**–**H** Immunofluorescence staining and quantitative analysis of γH2AX when PARP4 knockdown and overexpression after treatment with 1 μM CPT, 20 μM CDDP, or DMSO for 12 h. Scale bar = 20μm. **P < 0.01 and ***P < 0.001. ns, nonsignificant. **I** Immunofluorescence staining of PARP4, γH2AX, and DAPI in A2058 and FLFMM34 cells after treatment with 1 μM camptothecin, 20 μM cisplatin, or DMSO for 12 h. Scale bar = 20μm.

Since the low expression of PARP4 was significantly linked with defective DSB repair and genome instability (Fig. 1F and G), we speculated that PAPR4 may be involved in DSB repair. To demonstrate this, we assessed the expression of γH2AX, a marker of DSB, in melanoma cells following DNA damage induction. Western blot analysis revealed that neither PARP4 knockdown nor overexpression significantly affected γH2AX level. However, both cisplatin (CDDP) and camptothecin (CPT) could induce DSB, leding to a significant increase in γH2AX expression. Notably, PARP4 knockdown significantly elevated γH2AX expression, whereas PARP4 overexpression significantly decreased γH2AX expression upon DSB (Fig. 2A-D), suggesting that PARP4 is involved in DSB repair. Similar results were also observed in immunofluorescence experiments (Fig. 2E-H). Since PARP4 primarily located in the cytoplasm, we enquired about the mechanism behind PARP4 involvement in DSB repair. Next, we performed immunofluorescence staining to explore the association of PARP4 and γH2AX localization upon DSB. As shown in Fig. 2I, PARP4 could translocate into the nucleus upon DSB. Therefore, PARP4 translocates into the nucleus to participate in DSB repair when DSB occurs.

### PARP4 suppresses cell apoptosis and G2/M arrest upon DSB

**Fig. 3:**
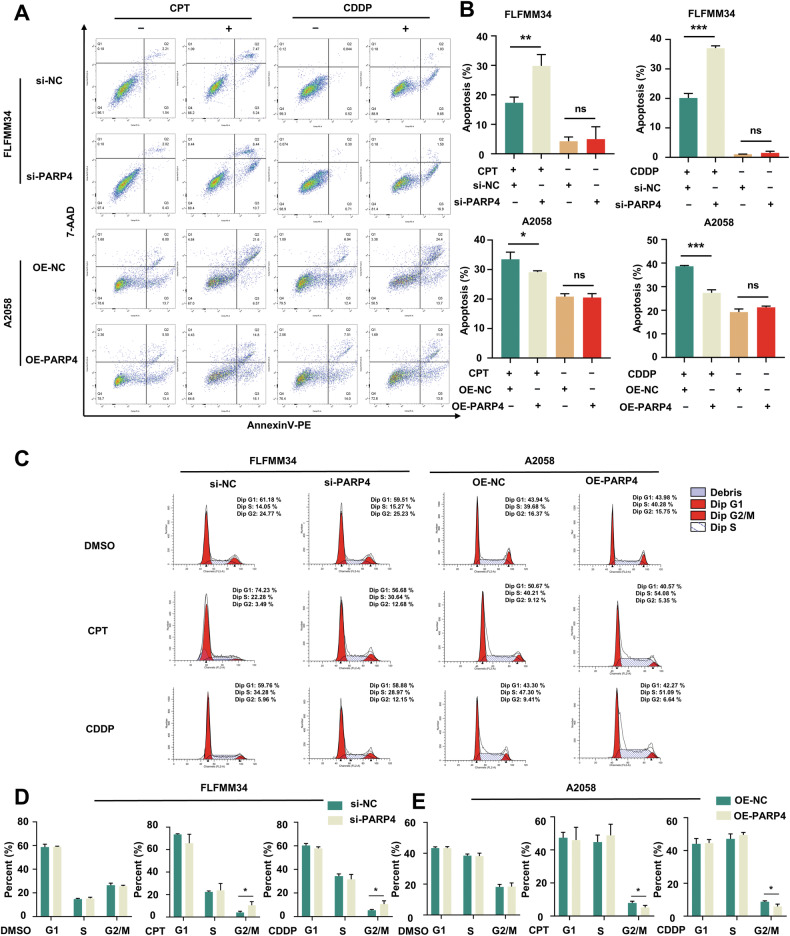
PARP4 suppresses melanoma cell apoptosis and G2/M arrest upon DSB. **A**, **B** Flow cytometry and quantitative analysis of cell apoptosis in FLFMM34 and A2058 cells when PARP4 knockdown and overexpression after treatment with 1 μM CPT, 20 μM CDDP, or DMSO for 24 h. The percentage of apoptosis is indicated. *P < 0.05, **P < 0.01 and ***P < 0.001. ns, nonsignificant. **C**–**E** Flow cytometry and quantitative analysis of cell cycle in FLFMM34 and A2058 cells when PARP4 knockdown and overexpression after treatment with 1 μM CPT, 20 μM CDDP, or DMSO for 24 h. The percentage of G1, S, and G2/M are indicated. *P < 0.05.

As accumulated DNA damage could lead to cell death by inducing cell apoptosis [12], Annexin V-PE/7AAD staining was performed to evaluate the effect of PARP4 on apoptosis. Flow cytometry indicated that PARP4 knockdown or overexpression showed no significant effect on apoptosis. But using the CDDP and CPT to induce DSB, the knockdown of PARP4 significantly enhanced cell apoptosis and PAPR4 overexpression showed the opposite effect (Fig. 3A and B). This indicated that PARP4 may involve in DSB repair to affect cell apoptosis.

DNA damage could also induce the cell cycle arrest, providing the cells with enough time to react. Thus, we evaluated the role of PARP4 in cell cycle. As shown in Fig. 3C–E, neither PARP4 knockdown nor overexpression had a significant impact on the cell cycle. Then we explored the cell cycle in both cell lines upon CDDP and CPT, which could induce stalled replication forks as well as cell cycle arrest [13, 14]. Consistent with previous studies [13, 14], we observed predominant S-phase arrest in two cell lines upon DSB (Fig. 3C–E). As illustrated in Fig. 3C–E, si-PARP4 and OE-NC presented longer G2/M phase, which indicated increased sensitivity to DNA-damaging agents as previous reports [14, 15].

### PARP4 participates in the NHEJ repair pathway by regulating the MARylation of Ku80

**Fig. 4:**
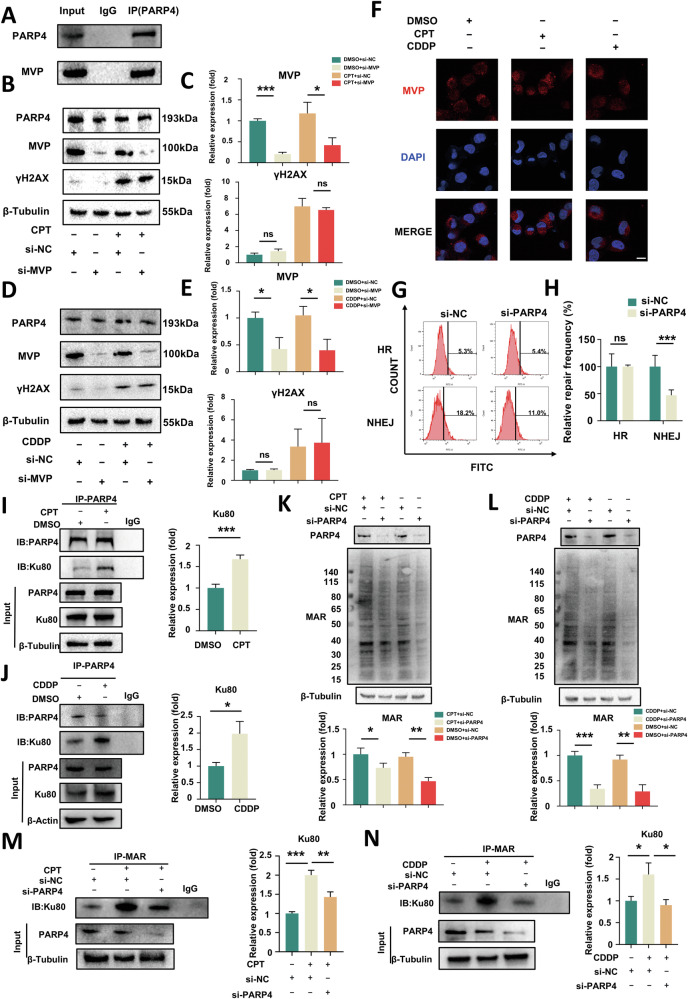
PARP4 participates in the NHEJ repair pathway by regulating the MARylation of Ku80 as a monomer. **A** Western blotting of PARP4 and MVP protein that was immunoprecipitated with anti-PARP4 antibodies for endogenous interaction. **B**–**E** Western blotting and quantitative analysis of changes of PARP4, MVP and γH2AX expression levels after administering 1 μM CPT or 20 μM CDDP to MVP knockdown FLFMM34 cells for 12 h. * *P* < 0.05, *** *P* < 0.001. ns, nonsignificant. **F** Immunofluorescence staining of MVP and DAPI in FLFMM34 cells upon 1 μM CPT or 20 μM CDDP treatment for 12 h. Scale bar = 20 μm. **G**, **H** Flow cytometry and quantitative analysis of the ratio of GFP+ cells in the si-NC and si-PARP4 groups. The average proportion of GFP+ cells in si-NC and si-PARP4 cell lines served as the measurement for HR or NHEJ repair. *** *P* < 0.001, ns, nonsignificant. FLFMM34 cells were exposed to DMSO, CPT (1uM) or CDDP (20uM) for 12 h. Then, cell extracts were immunoprecipitated using a control antibody (IgG), an anti-PARP4 antibody **I**, **J**, or an anti-mono-ADP-ribose binding reagent **M**, **N** and then it was analyzed with western blotting and quantified. * *P* < 0.05, ** *P* < 0.01 and *** *P* < 0.001. **K**, **L** Western blotting and quantitative analysis of expression levels of MAR in FLFMM34 cells when PARP4 knockdown after treatment with 1 μM CPT, 20 μM CDDP, or DMSO for 12 h. * *P* < 0.05, ** *P* < 0.01.

PARP4 has been identified as an interacting partner of MVP, involving the composition of the vault. To investigate whether PARP4 is involved in DSB repair as a monomer or a component of the vault complex, we first confirmed the interaction between MVP and PARP4 in FLFMM34 cells using coimmunoprecipitation (Co-IP) assays (Fig. 4A). Then, western blotting indicated that MVP knockdown did not affect γH2AX and PARP4 expression upon DSB (Fig. 4B–E). Additionally, immunofluorescence staining indicated that MVP was localized in the cytoplasm and it did not translocate into the nucleus upon DSB (Fig. 4F). These findings indicated that PAPR4 could be involved in DSB repair as a monomer independent of the vault.

To elucidate the specific pathway through which PARP4 contributes to DSB repair, we used DR-GFP and EJ5-GFP plasmids to monitor repair activity. These plasmids harbor I-SceI sites that generate I-SceI endonuclease-induced DSB. HR activity or NHEJ repair events can be detected by GFP+ cell proportion. We observed that NHEJ pathway repair efficiency significantly declined upon PARP4 knockdown. In contrast, HR pathway repair efficiency did not change (Fig. 4G and H), indicating that PARP4 is involved in DSB repair specifically through the NHEJ pathway. Previous studies have identified an interaction of PARP3 with Ku80, which plays a crucial role in NHEJ pathway by loading the NHEJ repair complex onto chromatin in response to DNA damage. Given that the structure of PARP4 is similar to PARP3, and that both are considered mono-ADP-ribosylation (MARylation) enzymes [16–18], we speculated that PARP4 could be involved in NHEJ repair pathway through a similar mechanism to PARP3. Firstly, we found that PARP4 interacted with Ku80, and the interaction enhanced upon DSB using Co-IP assays (Fig. 4I and J). Since Ku80 is primarily nuclear while PARP4 is predominantly cytoplasmic, this interaction likely reflected the translocation of PARP4 into the nucleus in response to DNA damage. Furthermore, we noted the level of MARylation decreased when PARP4 was knocked down (Fig. 4K and L), demonstrating the activity of MARylation of PARP4. Next, through Co-IP assays, the MARylation level of Ku80 increased upon DSB and it decreased dramatically when PARP4 was knocked down, suggesting that PARP4 was involved in the Ku80 MARylaytion (Fig. 4M and N). These data indicated that PARP4 participated in DSB repair as a monomer and it regulated Ku80 MARylation to participate in the NHEJ repair pathway.

### PARP4 deficiency enables melanoma to become more sensitive to ATM inhibitors in vitro and in vivo

**Fig. 5:**
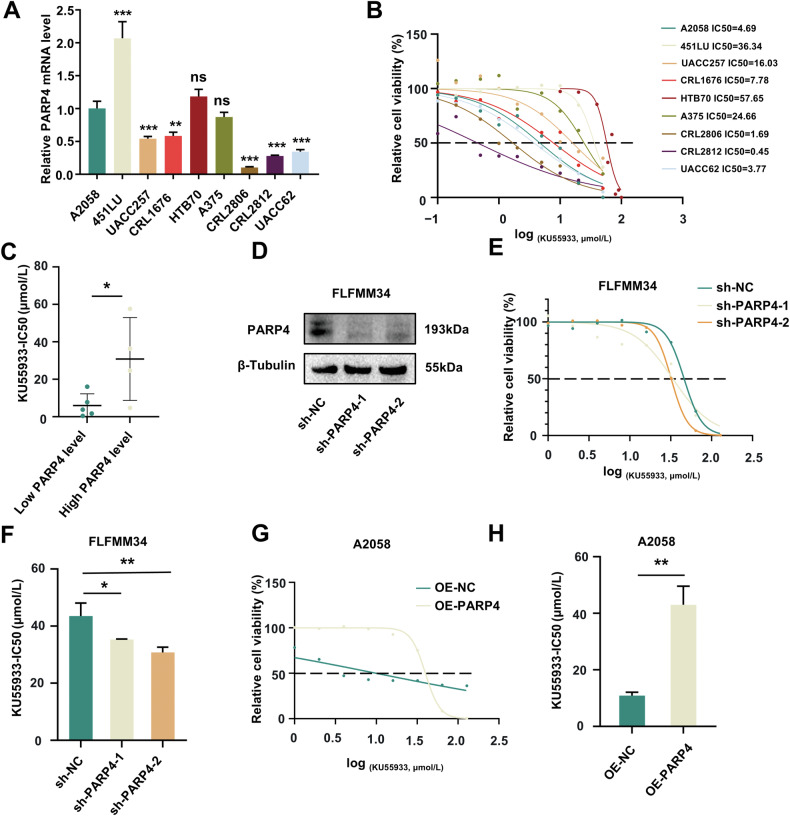
The cells with low PARP4 expression were more sensitive to KU55933. **A** PARP4 mRNA level was analyzed using qRT-PCR in different melanoma cell lines. **P < 0.01, ***P < 0.001, ns, nonsignificant. **B** CCK-8 assays helped measure the IC50 value for each melanoma cell line against KU55933. **C** The chart of the IC50 of cell lines with high PARP4 and low PARP4 levels. Low PARP4 expression was defined as cell lines exhibiting significantly lower levels than the A2058 cell line, while the others were classified as high expression. *P < 0.05. **D** The knockdown efficiency of PARP4 in FLFMM34 was analyzed using western blotting. **E** CCK-8 assays helped measure the IC50 value against KU55933 for PARP4 knockdown and control cells. **F** The bar chart of the IC50 of FLFMM34 cells with PARP4 knockdown and control cells. *P < 0.05, **P < 0.01. **G** CCK**-**8 assays helped measure the IC50 value against KU55933 for PARP4 overexpression and control cells. **H** The bar chart of the IC50 of A2058 cells with PARP4 overexpression and control cells. **P < 0.01.

Our results have suggested that the knockdown of PARP4 could reduce the repair efficiency of the NHEJ pathway, prompting us to investigate whether melanoma cells with low PARP4 expression were more sensitive to DNA damage repair inhibitors based on the synthetic lethal strategy. Half-maximal inhibitory concentration (IC50) is an important indicator for evaluating a patient’s response to drug therapy. Firstly, we used Genomics of Drug Sensitivity in Cancer (GDSC) data to predict the IC50 scores of different DNA damage repair inhibitors in 468 skin cutaneous melanoma. Among that, melanoma patients with low PARP4 expression (*N* = 234) were more sensitive to the ATM inhibitor KU55933 (Wilcoxon rank sum test, *P* = 0.043), but not to other inhibitors like ATR inhibitor (Wilcoxon rank sum test, dactolisib, *P* = 0.935; ceralasertib, *P* = 0.479; berzosertib, *P* = 0.57), or PARP inhibitor (Wilcoxon rank sum test, talazoparib, *P* = 0.336). These findings were validated by measuring PARP4 mRNA levels in various melanoma cell lines using RT-qPCR while testing the IC50 value of KU55933 in these melanoma cell lines through the cell viability assay (Fig. 5A and B). Low PARP4 expression was defined as cell lines exhibiting significantly lower levels than the A2058 cell line, while the others were classified as high expression. We noted that cell lines with low PARP4 expression levels were significantly more sensitive to KU55933 than those with high PARP4 expression (Fig. 5C). To further investigate this, two independent shRNAs targeting PARP4 were established against PARP4 in FLFMM34 cell lines (Fig. 5D). PARP4 knockdown and overexpression significantly affected the IC50 value of KU55933 in FLFMM34 and A2058 melanoma cell lines, with cells with low PARP4 expression had more sensitivity towards KU55933 (Fig. 5E–H).

**Fig. 6:**
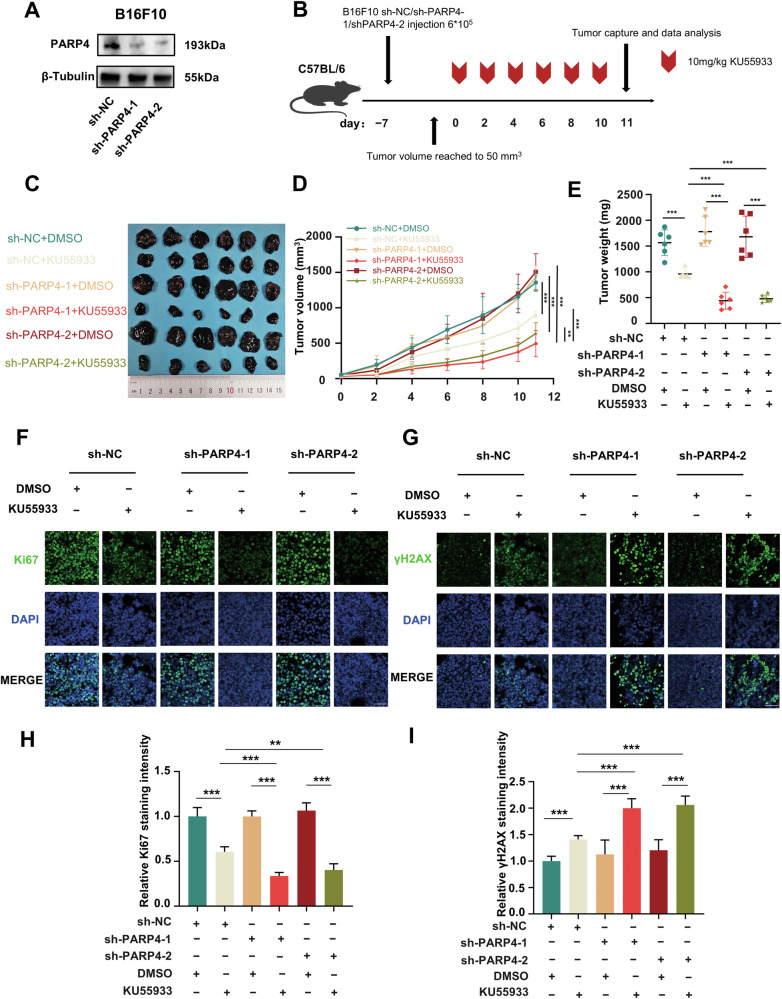
In vivo, the knockdown of PARP4 enhanced cell sensitivity to KU55933. **A** The knockdown efficiency of PARP4 in B16F10 was analyzed using western blotting. **B** A schematic view of the transplantation plan that C57BL/6 mice were burdened using sh-NC or sh-PARP4-1 or sh-PARP4-2 transfected B16F10 tumors. **C** The captured images of isolated tumors in xenografted mice receiving the indicated treatments. **D**–**E** Tumor volume and weight were measured for each group. **P < 0.01, ***P < 0.001, ns, nonsignificant. **F**, **G** Immunofluorescence staining of Ki67 and γH2AX in isolated xenograft tumors. **H**, **I** Quantitative analysis of Ki67 and γH2AX staining intensity in three isolated xenograft tumors. Each field contains at least 200 cells. Scale bar = 50 μm. **P < 0.01, ***P < 0.001.

Subsequently, a preclinical mice model was established to demonstrate that melanoma with low expression of PARP4 was more sensitive to KU55933 in vivo. This was achieved by establishing two independent shRNAs against PARP4 in B16F10 cell lines (Fig. 6A). Subsequently, the preclinical transplantation mouse model was established through subcutaneous inoculation of C57BL/6 mice with sh-NC, sh-PARP4-1, or sh-PARP4-2 modified B16F10 cells. After a week of tumor growth, the mice were treated with KU55933 or an equivalent volume of 5% DMSO through intraperitoneal injections on alternate days (Fig. 6B). Tumor volume and weight in groups sh-PARP4-1 and sh-PARP4-2 were comparable to sh-PARP4-NC. This indicated that the knockdown of PARP4 alone could not inhibit tumor progression. KU55933 monotherapy delayed tumor growth in the sh-PARP4-NC group. In contrast, tumor growth inhibition by KU55933 was more significant within the sh-PARP4-1 and sh-PARP4-2 groups (Fig. 6C–E). In addition, the staining intensity of Ki67 was reduced in the sh-PARP4-1 and sh-PARP4-2 groups treated with KU55933, while the staining intensity of γH2AX was increased in these tumors, indicating that DNA damage in these tumors was more severe (Fig. 6F–I). These data suggested that the tumors with low PARP4 expression were more sensitive to KU55933. Thus, KU55933 could be a promising treatment modality in PARP4 deficiency patients.

## Discussion

Our study highlighted the crucial role of PARP4 in DNA damage repair and its impact on the sensitivity of ATM inhibitor in melanoma therapy. Firstly, low PARP4 expression was significantly related to defective DSB repair, genome instability, and shorter DFS and OS in melanoma. Further validation confirmed that PARP4 participated in DSB repair, and when PARP4 was knocked down, melanoma cells failed to manage the overwhelming DNA damage, leading to cell apoptosis and cell cycle G2/M arrest. Subsequently, we demonstrated that PARP4 could interact with Ku80 and it regulated Ku80 MARylation to participate in the NHEJ repair pathway. Importantly, PARP4 knockdown enhanced melanoma cell sensitivity to ATM inhibitors in vitro and in vivo, suggesting the potential of exploiting synthetic lethality as a therapeutic strategy.

PARP protein family comprises 17 members (PARP1-16), which catalyze ADP-ribose modifications on target proteins using NAD+ as a substrate [19]. PARP4 is known for its co-composition with MVP, TEP1, and several small untranslated RNA molecules to form vault particle. A study observed that two PARP4 mutations were found in 43% of breast and thyroid cancer patients. In contrast, their frequencies were only 0.5% in controls. Low PARP4 level was linked with poorer prognosis in breast cancer [20]. Another study indicated that PARP4 knockdown significantly decreased the CDDP resistance [21]. Consistent with these studies, our study also demonstrated that PARP4 was linked with poorer prognosis in melanoma and the PARP4 knockdown significantly increased CDDP-induced cell apoptosis, suggesting that PARP4 may play a pivotal role in the occurrence, progression, and treatment of melanoma.

Recent studies indicated that PARP4 shared key domains with PARP3, including the WGR-HD-ART structural domain and catalytic triad (H-Y-E), suggesting a potential similarity in DNA damage repair mechanisms [16, 22]. Previous studies have reported that PARP3 performed DNA damage repair, primarily through the NHEJ pathway through interacting with Ku80 [23]. Therefore, we first demonstrated that PARP4 knockdown decreased NHEJ repair efficiency, indicating that PARP4 was involved in the NHEJ pathway, similar to PARP3. Then, we demonstrated that PARP4 interacted with Ku80, and it was involved in Ku80 MARylation to participate in the NHEJ repair pathway.

Recently, drug development targeting molecules essential to the DNA damage repair mechanism has been a hotspot in tumor drug research. Among them, synthetic lethal development has contributed significantly to the progression in this field. PARP Inhibitors were approved as first-generation synthetic lethal agents for advanced ovarian cancer with BRCA1 or BRCA2 mutations [24]. PARP inhibitors competitively bind to PARP, leading to many SSB within the cell. These SSB cannot be repaired in time and are transformed into DSB. In BRCA1/BRCA2-deficient tumor cells, the HR pathway is aberrant. A large number of DSB cannot be repaired through the HR pathway but through other error-prone pathways, leading to tumor cell death. We have demonstrated that PARP4 was involved in the NHEJ pathway. Targeted molecular inhibition in the HR repair pathway in PARP4-low expression melanoma could lead to synthetic lethality and therapeutic effects. ATM, a core component of the DNA repair system, is activated to improve the HR repair pathway upon DSB [25]. Our study demonstrated that melanoma with low PARP4 expression was more sensitive to KU55933 in vitro and in vivo. Currently, ATM inhibitor AZD0156 is assessed in phase I clinical trials (NCT02588105). Our study provides an essential reference for the clinical application of KU55933 in melanoma, along with a theoretical basis for clinical individualized treatment.

In conclusion, our findings provide novel insights into the functional role of PARP4 in DNA damage repair and melanoma pathogenesis. Moreover, our study also suggests that the ATM inhibitor may represent a potential therapeutic approach for treating melanoma with low PARP4 expression, offering a potential avenue for individualized treatment strategies.

## Materials and Methods

### Public database analysis

The expressions of PARP4 genes in clinical samples were displayed with the Human Protein Atlas (HPA) (https://www.proteinatlas.org/). GEPIA 2.0 (Gene Expression Profiling Interactive Analysis) (http://gepia2.cancer-pku.cn/) was used to acquire the PARP4 genes’ expression profiles between normal and melanoma tissues, the disease-free survival, and the overall survival. TAI and HRD_sum values of melanoma samples were analyzed using the TCGA database. Using publicly available pharmacogenomics database, Genomics of Drug Sensitivity in Cancer (GDSC), the sample-based transcriptome predicts the response of each sample in TCGA database to the different DNA damage repair inhibitors of melanoma.

### Cell lines

FLFMM34 was a metastatic melanoma cell line developed in our lab [26]. A375, CRL1676, and mouse melanoma cell line B16F10 were grown in DMEM (Hyclone, #SH30285.FS) supplemented with 10% FBS. A2058, FLFMM34, CRL2806, and CRL2812 were grown in DMEM/F12 (Hyclone, #SH30023.02) supplemented using 10% FBS. UACC62, UACC257, 451 LU, and HTB70 were cultured in RPMI1640 medium (Hyclone, #SH30027.01) supplemented with 10% FBS. All the cells were maintained at 37 °C inside an incubator using humidified 5% CO_2_ gas.

### Chemicals and antibodies

The chemicals and antibodies utilized in this study are listed in Supplementary Methods.

### qRT-PCR

Total RNA was extracted with the TRIzol reagent (Invitrogen, Scotland, UK, #15596018). Reverse transcription was performed using a High Capacity cDNA Reverse Transcription Kit (TaKaRa, Kyoto, Japan, #RR036A). Quantitative PCR was conducted with the SYBR Premix Ex Taq II kit (TaKaRa, #RR036A) on a QuantStudio 6 Flex Real-Time PCR System (Hercules, CA, USA). The 2^-ΔΔCt^ method helped quantify gene expression normalized to β-actin mRNA. The primers utilized in this study were: β-Actin: Forward (F) 5′-CCTGGCACCCAGCACAAT-3′, Reverse(R) 5′-GGGCCGGACTCGTCATAC-3′; PARP4: Forward (F) 5′- GTGAACAGGATTAGCCTCAACG-3′, Reverse(R) 5′-TCTTAGCCAATAGTCCCAGGTT′.

### Plasmid vectors, RNA Interference and gene transfection

The PARP4 overexpression plasmid (ORIGENE, Maryland, USA, #RC220444) and the according control plasmid pCMV6-Entry (ORIGENE, #PS100001) were purchased from ORIGENE. Small interfering RNAs were procured from Tsingke (Beijing, China). The small interference RNA sequences are si-PARP4: 5’ -AAACAAGGAUUUCUACUAAGA-3’and si-MVP: 5’-CUGCUGUCUUUGGCUUUGATT-3’. Lipofectamine 3000 (Invitrogen, # L3000015) reagent was used as per the manufacturer’s protocol. The transfection of FLFMM34 cells was performed using the small interference RNAs. PARP4 lentivirus shRNA were procured from Tsingke. Human PARP4 lentivirus sequences are shRNA-1, 5’-CCTGGGACTATTGGCTAAGAACTCGAGTTCTTAGCCAATAGTCCCAGGTTTTTT-3’; shRNA-2, 5’- GCATTCAATCTCTAGGTGTAACTCGAGTTACACCTAGAGATTGAATGCTTTTTT-3’. Mouse PARP4 lentivirus sequences are shRNA-1, 5’-GCTTGAAGGTCAAGTACTTACCTCGAGGTAAGTACTTGACCTTCAAGCTTTTTT-3’; shRNA-2, 5’-GGTGTCATCTGAACTCCATCCCTCGAGGGATGGAGTTCAGATGACACCTTTTTT-3’. The negative control sequences are shRNA-NC, 5’- GATCCACTACCGTTGTTATAGGTGTTCAAGAGACACCTATAACAACGGTAGTTTTTTTG-3’. The FLFMM34 and B16F10 cells were infected with lentivirus supernatant for 48 h and selected with puromycin (1 μg/ml) for 8 days to get the stable pooled clones.

### Immunofluorescence assay

Cells were fixed with 4% paraformaldehyde for 15 min at room temperature. This was followed by permeabilization using 0.2% Triton X-100 in PBS for 10 min. After blocking using 5% goat serum for 1 h, cells were incubated with primary antibodies overnight at 4 °C. After PBS washing, cells were incubated using fluorophore-conjugated secondary antibodies for 1 h at room temperature. Nuclei were counterstained using Hoechst 33258 (1:1000, Beyotime, Shanghai, China, #C1011) for 15 min. The stained cells were observed with a Zeiss LSM880 confocal laser scanning microscope.

### Cell viability assay

The cells were plated at 3,000 per well in 96-well plates and incubated at 37 °C with 5% CO_2_ and humid conditions. The fresh medium comprising 1/10 CCK-8 reagent was added for incubation for 90 min at 37 °C with 5% CO_2_. Then, the cell viability was assessed by determining absorbance at 450 nm.

### Apoptosis and cell cycle analysis

Cells were washed using phosphate buffer saline PBS, resuspended at a 1 × 10^6^ cells/mL density, and assessed for apoptosis with the Annexin PE/7-AAD kit from Vazyme (#A213-01) based on the manufacturer’s instructions. Then, a Coulter FC500 flow cytometer from Beckman was used for analysis. For cell cycle analysis, 1×10^6^ cells were washed, fixed in 70% ethanol overnight, and evaluated using the Cell Cycle and Apoptosis Analysis Kit from Beyotime (#C1052) according to the manufacturer’s instructions. Then, it was analyzed using a Coulter FC500 flow cytometer from Beckman.

### Western blotting

Proteins were extracted and quantified from the cell lysates. Equal protein (20-30 μg) amounts were separated using sodium dodecyl sulfate-polyacrylamide gel electrophoresis (SDS-PAGE) and transferred onto polyvinylidene fluoride (PVDF) membranes. The membranes were blocked using 5% non-fat milk in Tris-buffered saline with Tween-20 (TBST) and incubated overnight at 4 °C using primary antibodies specific to the target protein. After TBST washing, membranes were incubated using horseradish peroxidase (HRP)-conjugated secondary antibodies at room temperature for 1 h. Then, membranes were washed again using TBST. Protein bands were visualized with an enhanced chemiluminescence (ECL) substrate.

### HR and NHEJ reporter assay

The pimEJ5GFP or pDRGFP plasmids (Tsingke Biotechnology, China) were employed to transfect cells using the Lipofectamine 3000 reagents. Puromycin screening ensured that the DR-GFP and EJ5-GFP construct was expressed. Then, the I-SceI expression vector was transiently transfected (Tsingke Biotechnology, China) using Lipofectamine 3000 reagents. At 36 hours after transfection, the BD LSRFortessa Flow Cytometer was applied to determine the GFP signal.

### Co-Immunoprecipitation

Details for Co-Immunoprecipitation are described in Supplementary Methods.

### Xenograft mice model and treatments

Details for Xenograft mice model and treatments are described in Supplementary Methods.

### Statistical Analysis

All the data represent the mean ± SD from at least three independent experiments. The Log-rank test helped compare survival differences between groups. Statistical comparisons were conducted using the two-tailed Student’s t-test. All the data were analyzed by GraphPad Prism 8.0, and the *P* value < 0.05 (*) was considered statistically significant.

## Supplementary information

Supplementary Figure

Supplementary Methods

Western blot original data

## Data Availability

All the data can be obtained by contacting the corresponding author.
