# Supplementary material for: PARP4 deficiency enhances sensitivity to ATM inhibitor by impairing DNA damage repair in melanoma
Source: Cell Death Discov. 2025 Jan 30;11:35. doi: 10.1038/s41420-025-02296-0 (PMC11782537; doi:10.1038/s41420-025-02296-0)
Supplement: Supplementary file 1 — Supplementary Figure [file 41420_2025_2296_MOESM1_ESM.docx]

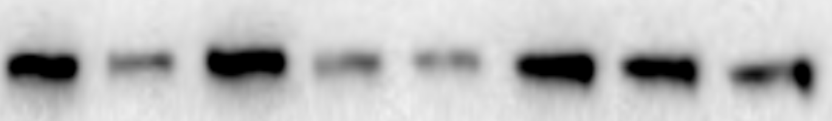

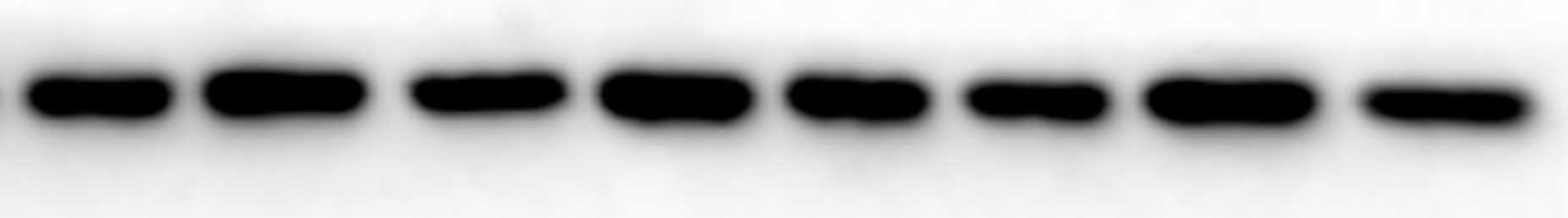


**193kDa**

**PARP4**

**GAPDH**

**36kDa**

**WM35**

**A375**

**451LU**

**HTB67**

**UACC257**

**FLFMM34**

**UACC62**

**A2058**

**
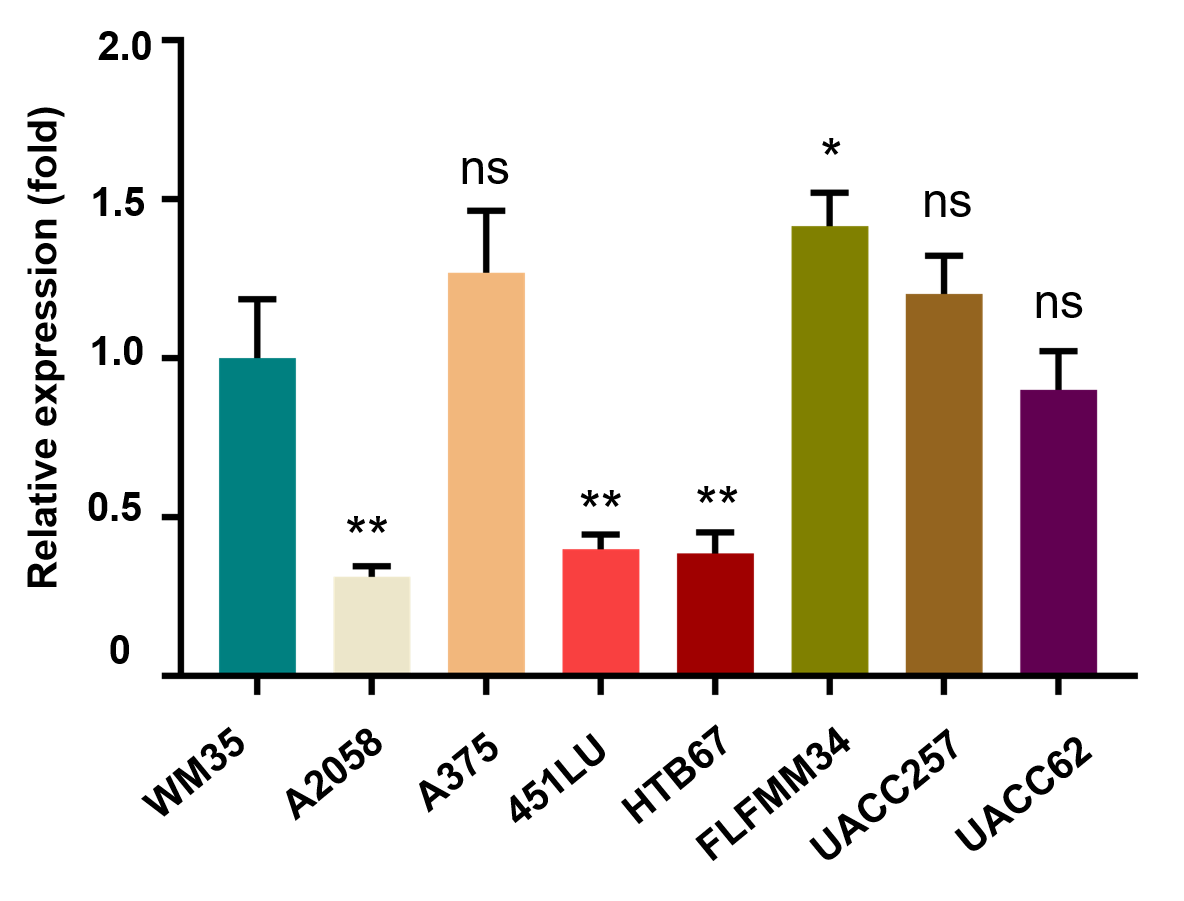
**

**Supplementary Figure 1. The expression of PARP4 within the different cell lines.**

Western blotting and quantitative analysis of the expression of PARP4 within the different melanoma cell lines.


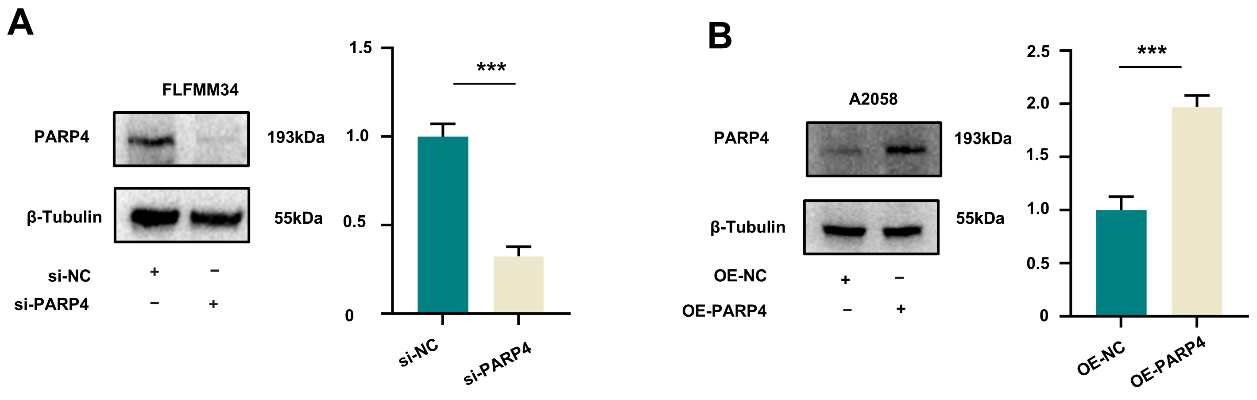


**Supplementary Figure 2. The knockdown and overexpression efficiency of PARP4 within FLFMM34 and A2058 cell lines. (A-B)** Western blotting and quantitative analysis of the knockdown and overexpression efficiency of PARP4 within FLFMM34 and A2058 cell lines.


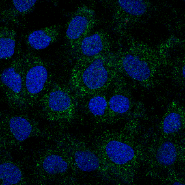

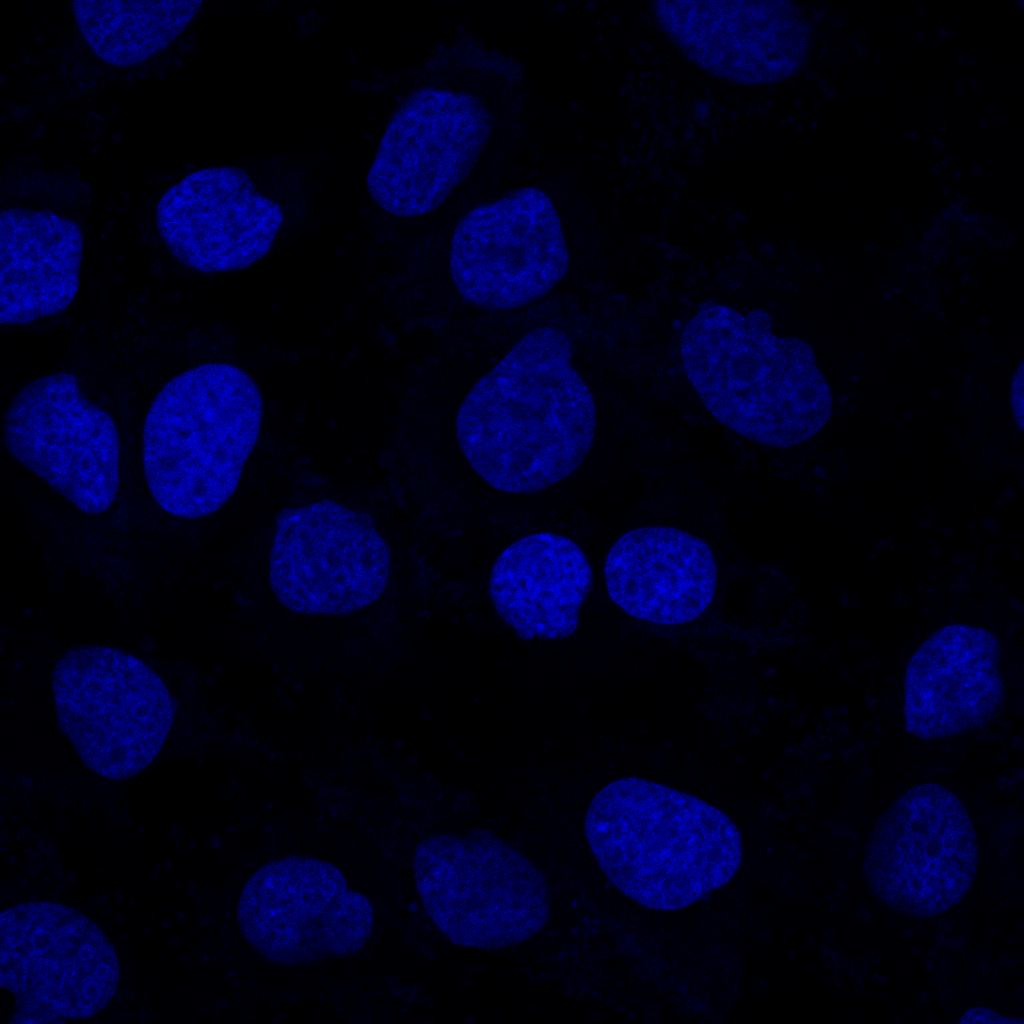

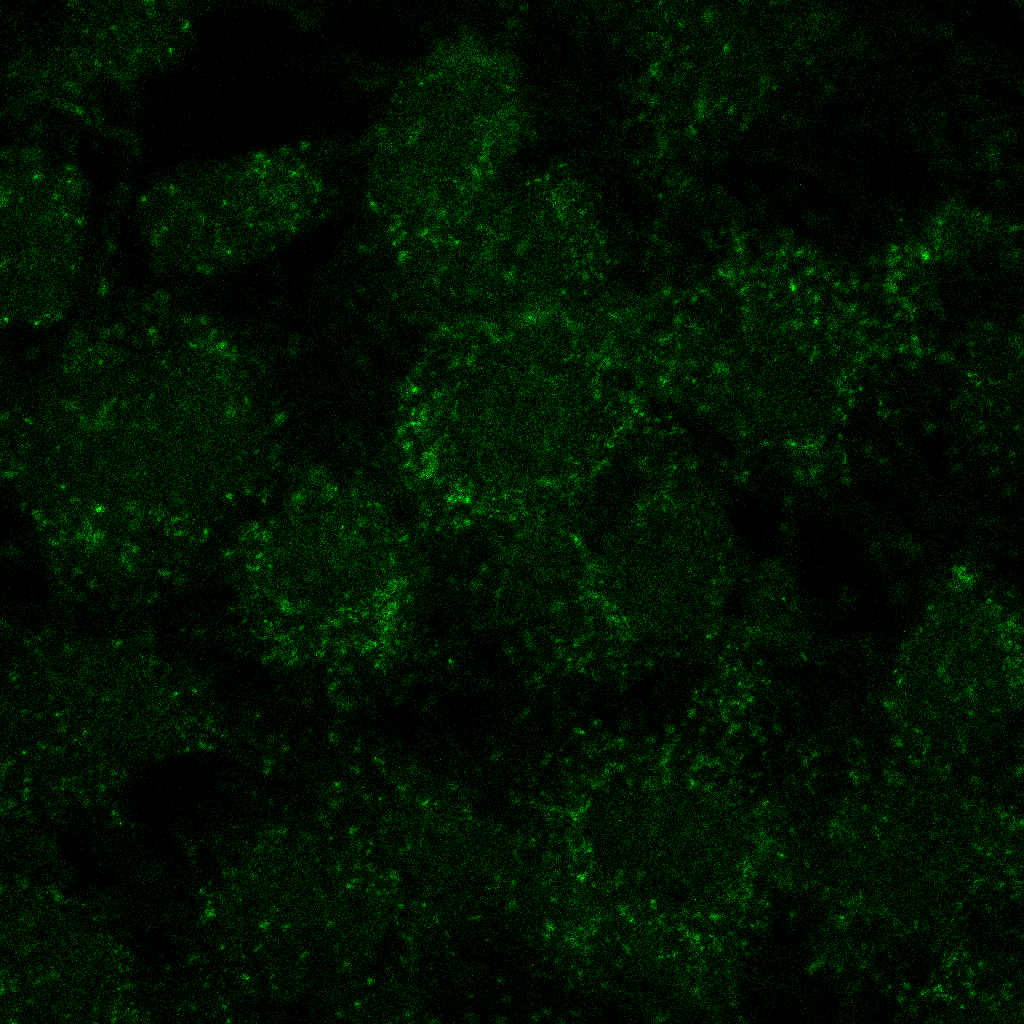

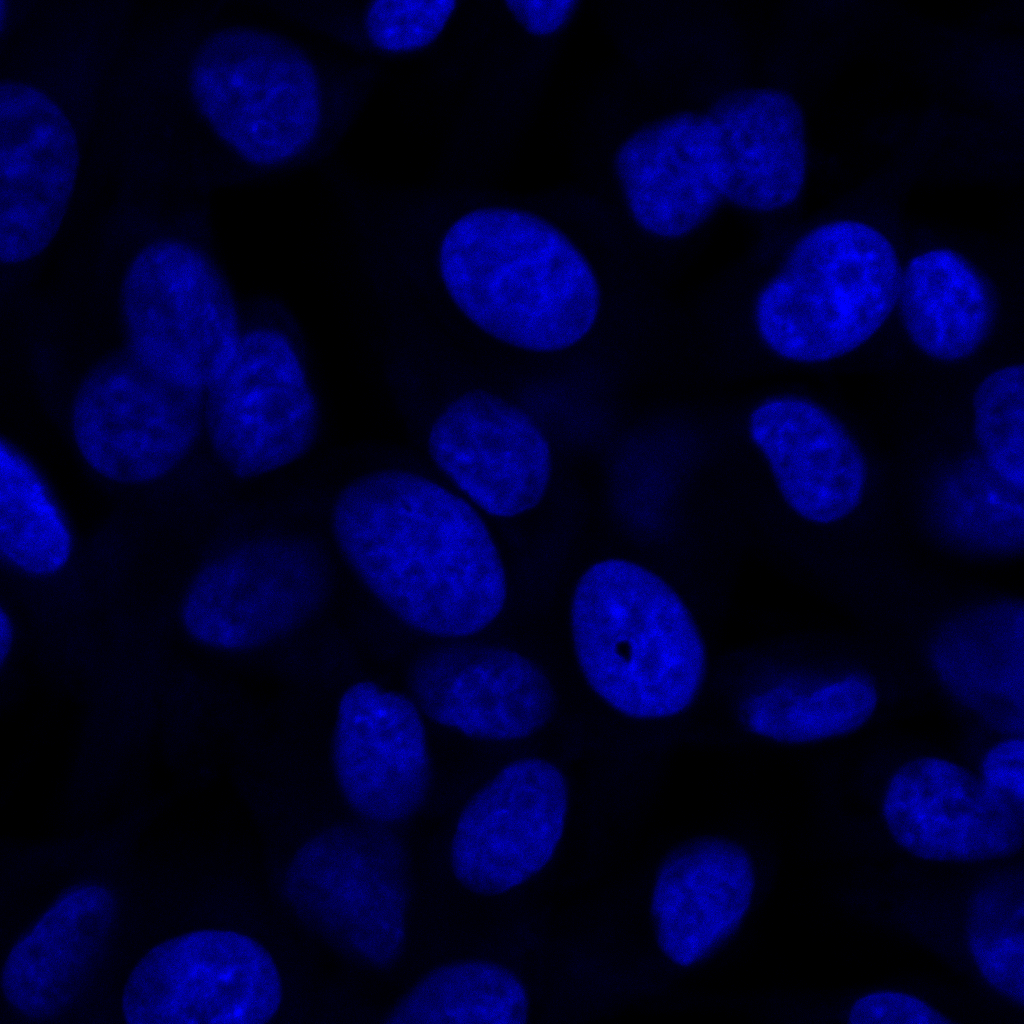

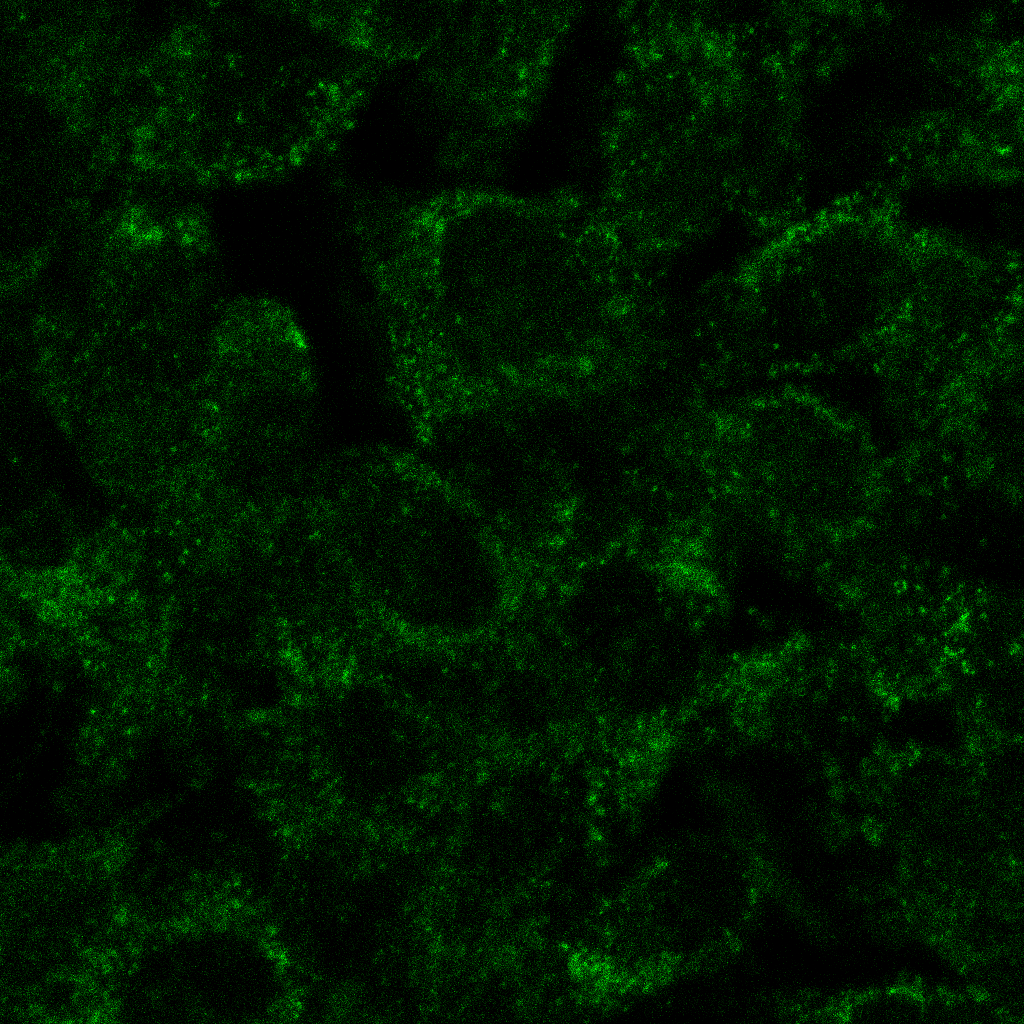


**PARP4**

**DAPI**

**MERGE**

**PARP4**

**DAPI**

**MERGE**

**FLFMM34**

**A2058**


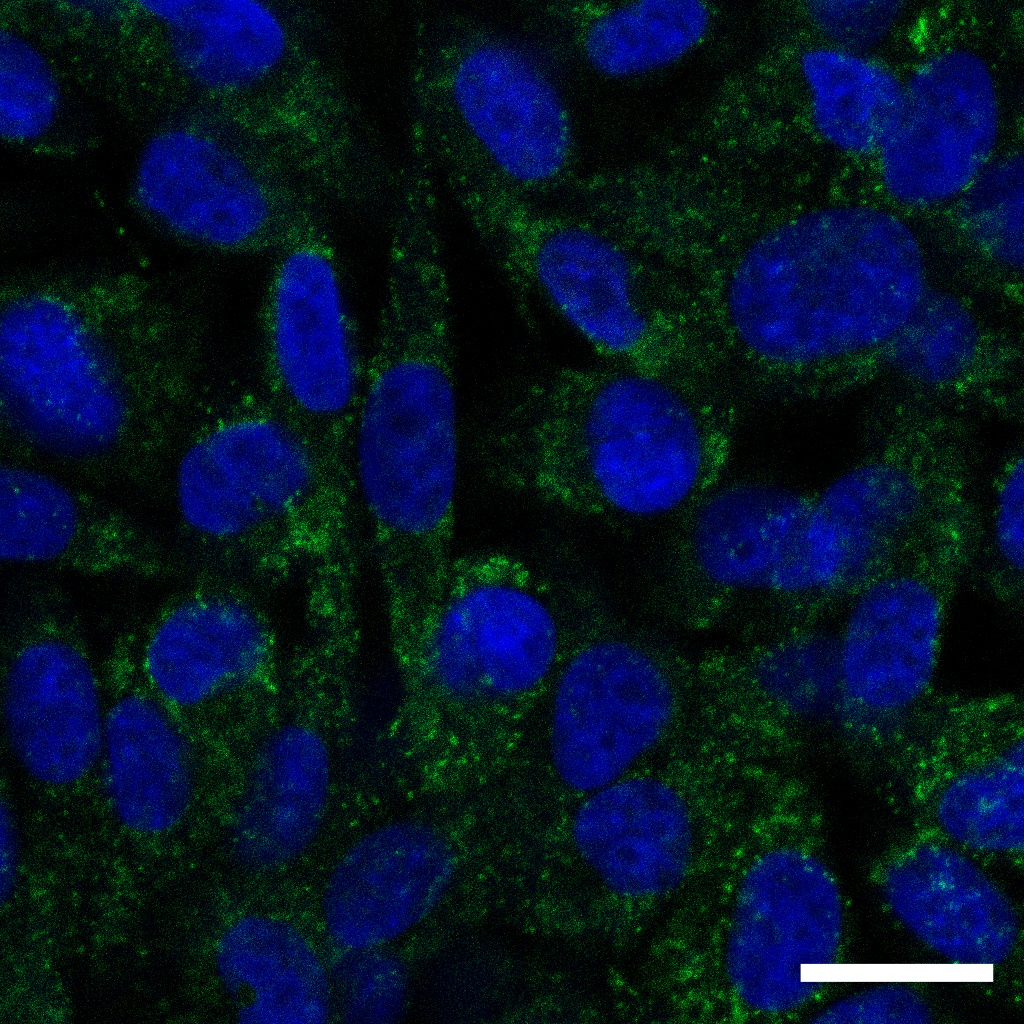


**A**

**B**

**Supplementary Figure 3. The distribution of PARP4 within the FLFMM34 and A2058 cells. (A-B)** Immunofluorescence staining of PARP4 and DAPI in A2058 and FLFMM34 cells. Scale bar = 20μm.
