# Supplementary material for: PARP4 deficiency enhances sensitivity to ATM inhibitor by impairing DNA damage repair in melanoma
Source: Cell Death Discov. 2025 Jan 30;11:35. doi: 10.1038/s41420-025-02296-0 (PMC11782537; doi:10.1038/s41420-025-02296-0)
Supplement: Supplementary file 2 — Supplementary Methods [file 41420_2025_2296_MOESM2_ESM.docx]

**Supplementary Methods**

**Chemicals and** **antibodies**

The chemicals and antibodies utilized in this investigation are listed below: camptothecin (Solarbio, Beijing, China, #SC8970), cisplatin (Solarbio, #D8810), and KU55933 (Selleck, Houston, TX, USA, #S1092). The primary and secondary antibodies used in Immunofluorescence assay were rabbit anti-PARP4 (1:150, Dallas, USA, #sc-515898), rabbit anti-γH2AX (1:200, Cell Signaling Technology, Massachusetts, USA, #9718), rabbit anti-Ki67 (1:200, Proteintech, Wuhan, China, #27309-1-AP), mouse anti-MVP (1:100, biorbyt, Cambridge, UK, #orb388964), goat anti-rabbit IgG H&L (FITC), (1:150, Zhuangzhibio, Xi’an, China #EK023), goat anti-mouse IgG H&L (Cy3), (1:150, Zhuangzhibio, #EK012). The primary and secondary antibodies used in western blotting were Primary antibodies against PARP4 (1:1000, Abcam, #ab133745), Beta-Actin (1:5000, Proteintech, #20536-1-AP), Beta-Tubulin (1:5000, Proteintech, #10094-1-AP), γH2AX (1:1000, Cell Signaling Technology, #9718), MVP (1:1000, Abcam, Cambridge, UK, #ab175239), Ku80 (1:1000, Proteintech, #16389-1-AP), and anti-mono-ADP-ribose binding reagent (8μg/ml, MilliporeSigma, Darmstadt, Germany, MABE1076).

**Co-Immunoprecipitation**

The cells were lysed using a lysis buffer containing protease inhibitors to obtain total protein extracts. The protein extracts were incubated with 5μg PARP4 antibody (Biomol, Hamburg, Germany, #A301-954A), 5μg anti-mono-ADP-ribose binding reagent (MilliporeSigma, Darmstadt, Germany, MABE1076), or 3μg IgG antibody (Proteintech, Wuhan, China, #30000-0-AP) for 2 hours at 4°C. Protein A/G beads (Santa Cruz Biotechnology, #sc-2003) were added to the antibody-protein mixture and incubated at 4°C overnight for binding. The beads are washed with PBST to remove unbound proteins. The protein-antibody complexes are eluted from the beads with an SDS loading buffer and boiled for 10 minutes. The yielded precipitates were analyzed using immunoblotting with anti-PARP4 (1:1000, Abcam, #ab133745) and anti-Ku80 (1:1000, Proteintech, #16389-1-AP).

**Xenograft mice model and treatments**

After one week of acclimatization, six-week-old female C57BL/6 mice (18-22 g) were randomly divided into three groups. 6 × 10^5^ B16F10-shNC/ B16F10-shPARP4-1/ B16F10-shPARP4-2 cells were diluted in 150 μl PBS and injected subcutaneously to develop subcutaneous tumors. When the tumor size reached 50 mm^3^, these groups were treated using 5% DMSO or 10mg/kg KU55933. Then, the tumor growth of mice was monitored by quantifying tumor length (L) and width (W) (tumor volume = L × W^2^/2). KU55933 was injected intraperitoneally into mice for four days (10mg/kg/day). After the indicated time, the mice were sacrificed. Then, the tumors were harvested and photographed, and tumor weights were examined. Measurements and data processing were performed blindly. For immunofluorescence staining analysis, tumors were stabilized in 4% paraformaldehyde overnight and subjected to embedding in paraffin. The animal experiments complied with ethical regulations and were approved by the Subcommittee on Research Animal Care of the Fourth Military Medical University (Xi'an, Shaanxi, China).
