# Supplementary material for: PARP4 deficiency enhances sensitivity to ATM inhibitor by impairing DNA damage repair in melanoma
Source: Cell Death Discov. 2025 Jan 30;11:35. doi: 10.1038/s41420-025-02296-0 (PMC11782537; doi:10.1038/s41420-025-02296-0)

Fig. 2A

IB: PARP4

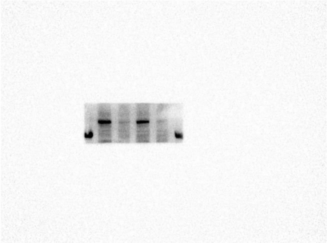

IB: γH2AX

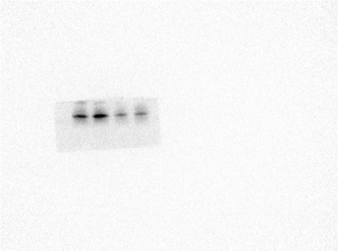

IB: β-Tubulin

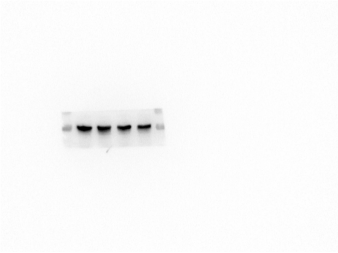

Fig. 2B

IB: PARP4

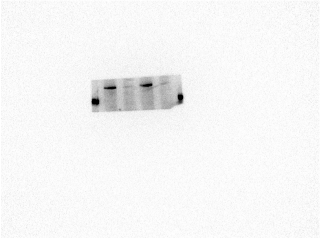

IB: γH2AX

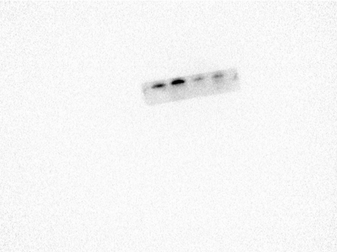

IB: β-Tubulin

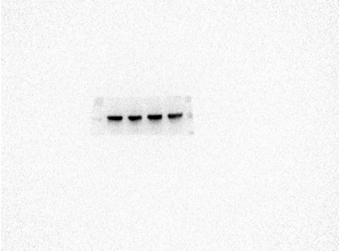

Fig. 2C

IB: PARP4

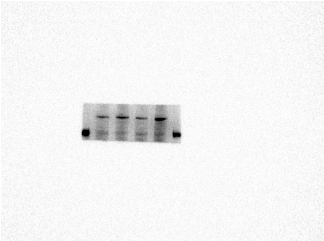

IB: γH2AX

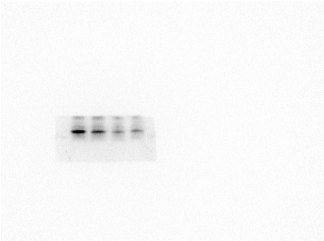

IB: β-Tubulin

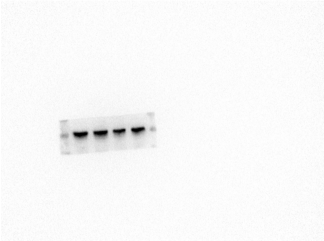

Fig. 2D

IB: PARP4

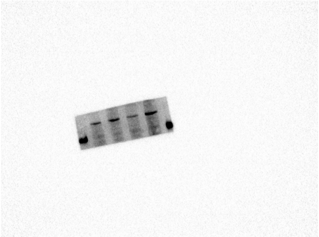

IB: γH2AX

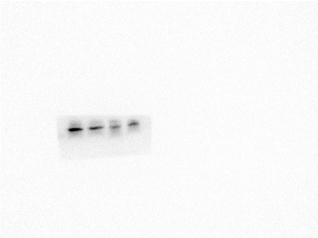

IB: β-Tubulin

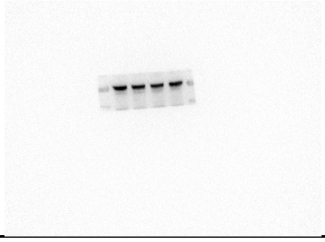

Fig. 4A

IB: PARP4

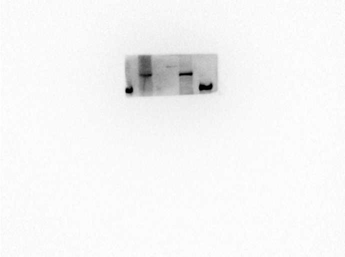

IB: MVP

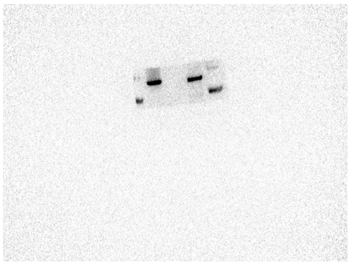

Fig. 4B

IB: PARP4

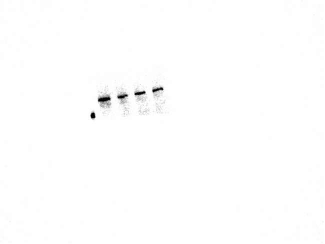

IB: MVP

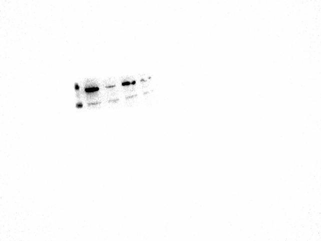

IB: γH2AX

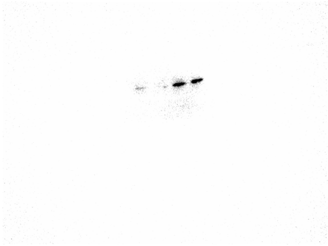

IB: β-Tubulin

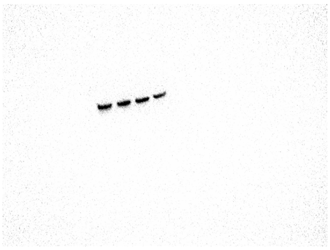

Fig. 4D

IB: PARP4

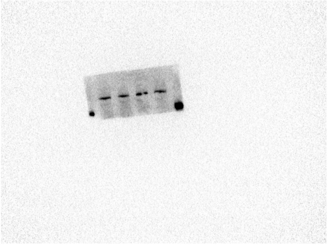

IB: MVP

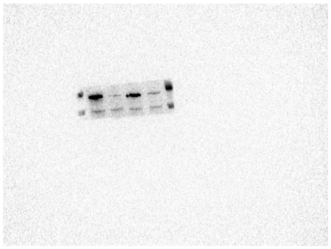

IB: γH2AX

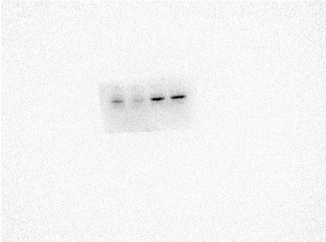

IB: β-Tubulin

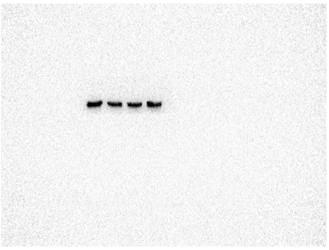

Fig. 4I

IB: PARP4

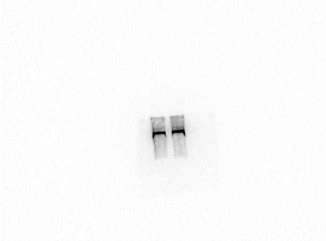

IB: Ku80

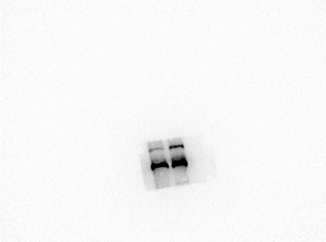

IB: PARP4

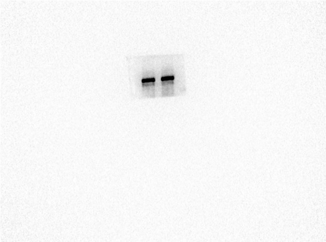

IB: Ku80

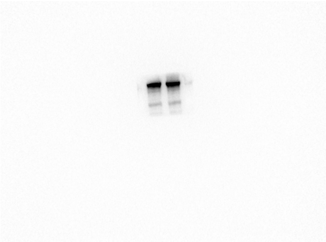

IB: β-Tubulin

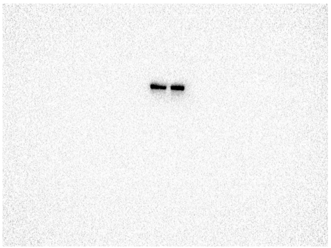

Fig. 4J

IB: PARP4

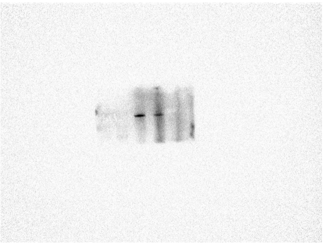

IB: Ku80

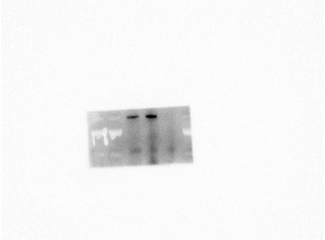

IB: PARP4

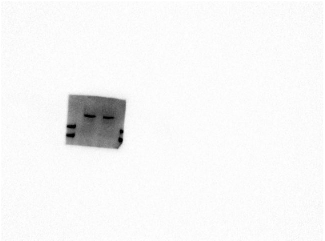

IB: Ku80

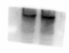

IB: β-Tubulin

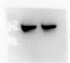

Fig. 4K

IB: PARP4

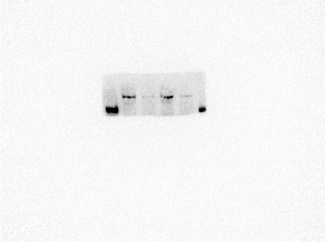

IB: MAR

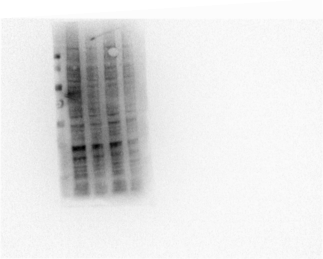

IB: β-Tubulin

Fig. 4L

IB: PARP4

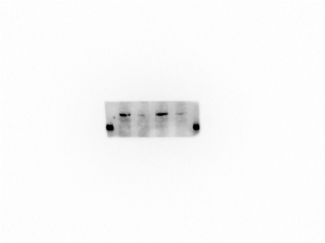

IB: MAR

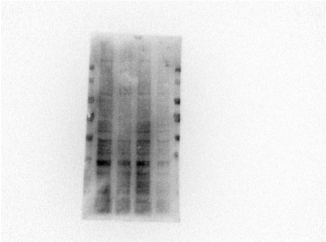

IB: β-Tubulin

Fig. 4M

IB: Ku80

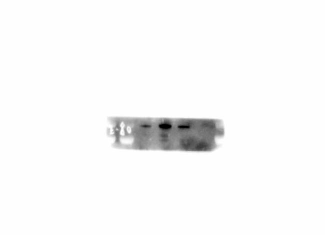

IB: PARP4

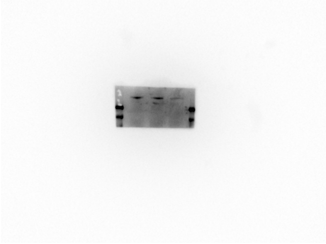

IB: Ku80

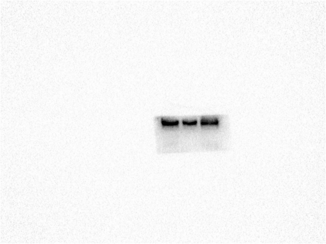

Fig. 4N

IB: Ku80

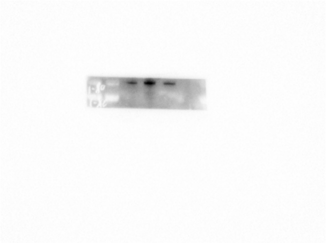

IB: PARP4

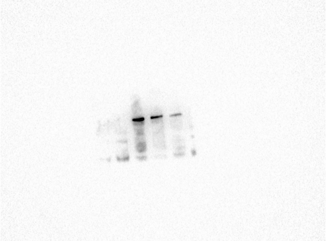

IB: Ku80

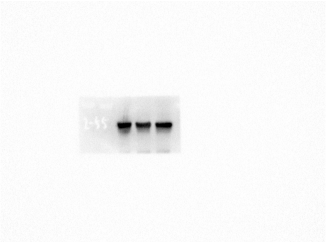

Fig. 5D

IB: PARP4

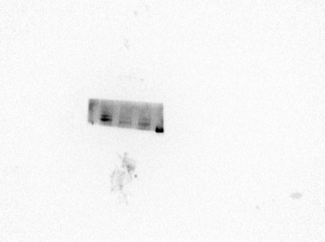

IB: β-Actin

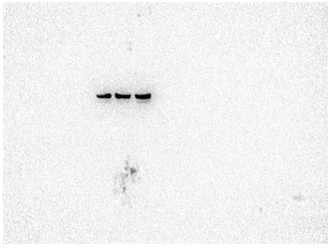

Fig. 6A

IB: PARP4

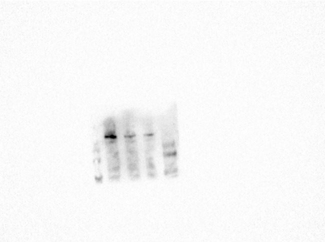

IB: β-Tubulin

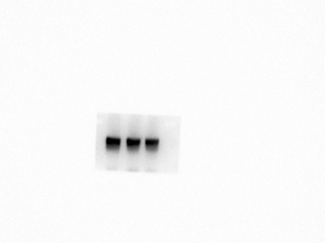

Supplementary Figure. 1

IB: PARP4

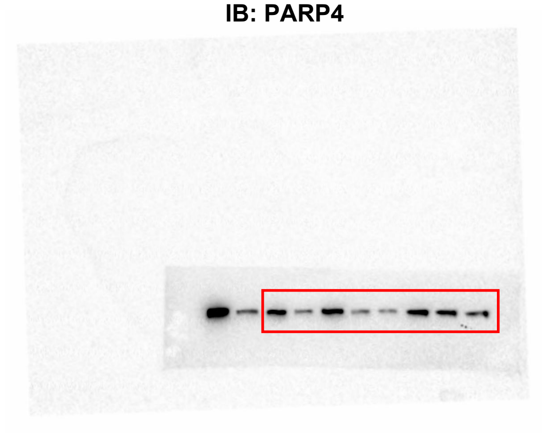

IB: GAPDH

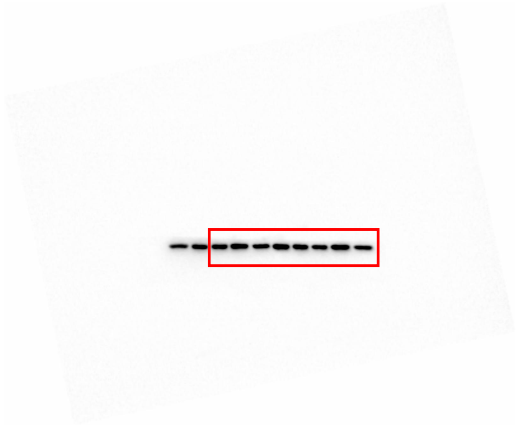

Supplementary Figure. 3A

IB: PARP4

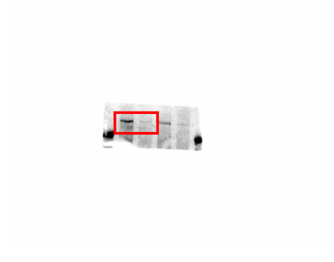

IB: β-Tubulin

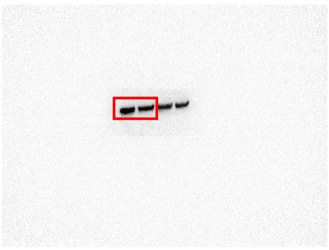

Supplementary Figure. 3B

IB: PARP4

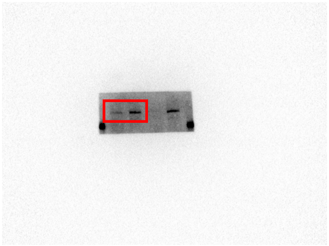

IB: β-Tubulin

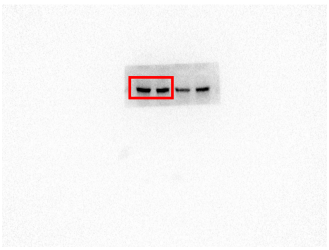

Supplement: Supplementary file 3 — Western blot original data [file 41420_2025_2296_MOESM3_ESM.pdf]
